# Mental health assessments in refugees and asylum seekers: evaluation of a tablet-assisted screening software

**DOI:** 10.1186/s13031-017-0120-2

**Published:** 2017-10-02

**Authors:** Naser Morina, Simon M. Ewers, Sandra Passardi, Ulrich Schnyder, Christine Knaevelsrud, Julia Müller, Richard A. Bryant, Angela Nickerson, Matthis Schick

**Affiliations:** 1 Department of Psychiatry and Psychotherapy, University Hospital Zurich, University of Zurich, Culmannstrasse 8, CH-8091 Zurich, Switzerland; 2 Department of Clinical Psychological Intervention, Freie Universität, Berlin, Germany; 3 Psychiatric Services Thurgau, CH-8596 Münsterlingen, Switzerland; 4 School of Psychology, University of New South Wales, Sydney, N.S.W. 2052 Australia

**Keywords:** Refugees, ACASI, Interview, Screening, Clinical setting, Usability, Feasibility

## Abstract

**Background:**

Mental health problems resulting from persecution and forced migration are very common among refugees and asylum seekers and evolve into a major public health challenge in hosting societies. Language barriers often prevent timely access to appropriate health care, leading to chronic trajectories and abortive social integration. Tools for multilingual screening and assessment could be of great benefit for this particularly vulnerable population as well as for policy makers. This study aimed at testing the reliability, feasibility and usability of the Multi-Adaptive Psychological Screening Software (MAPSS), a newly developed Audio Computer-Assisted Self-Interview Software (ACASI) for touchscreen devices, for screening purposes in a clinical setting.

**Methods:**

In a randomized cross-over design including both MAPSS and paper-pencil clinician-administered interviews, 30 treatment-seeking refugees completed clinical measures and a feasibility questionnaire to rate the user interface of MAPSS. Five professionals performed given tasks in MAPSS and completed usability questionnaires for the administration interface.

**Results:**

Results showed no differences between the two assessment modalities with regard to symptom scores. The findings suggest good feasibility and usability of MAPSS in traumatized refugees. The administration via MAPSS was significantly shorter than the paper-pencil interview.

**Conclusion:**

MAPSS may be a cost-effective, flexible and valid alternative to interpreter-based psychometric screening and assessment.

## Background

The United Nation High Commissioner for Refugees (UNHCR) reported that by the end of 2016 approximately 65.6 million people were forcibly displaced due to human rights violations, persecution, conflict, and organized violence [1]. The total number of refugees reached 21.3 million by mid-2015, its highest level since World War II [2]. By virtue of a wide range of traumatic and post-migration stressors, refugees are at high risk for severe mental health issues [3, 4]. High prevalence rates for Posttraumatic Stress Disorder (PTSD; 30.6%) and depression (30.8%) were reported in a meta-analysis of 181 studies with a total of over 80,000 war-affected refugees [5]. Another meta-analysis reported similar findings in adult war-refugees 5 years or longer after displacement, pointing to the high prevalence of chronic trajectories [6].

Efficient and evidence-based treatment options for trauma related and other mental health problems exist [7, 8] and are in principle available in many hosting societies. In addition to distrust, shame, stigma, and/or the lack of knowledge about psychological disorders and treatment possibilities, however, language and communication difficulties often prevent refugees from accessing health care and treatment post-resettlement [9]. While guidelines recommend the use of qualified interpreters when providing treatment to refugees [10, 11], the coverage of costs is often not warranted and manageable neither by patients nor by service providers. As a result, the use of lay or ad hoc interpreters such as family members is commonly practiced, although the literature has evidenced manifold disadvantages regarding disclosure of sensitive information/confidentiality, communication (errors and comprehension), utilization, clinical outcomes and satisfaction with care [12–14]. Ultimately, lack of proficiency in the host country’s language represents a substantial barrier to the identification and treatment of health problems in refugees (e.g. [15]).

Whereas qualified interpreters are indispensable to the therapeutic process, computer-based tools can be effectively implemented in screening and diagnostic procedures in terms of standardized psychological assessments. In comparison to face-to-face interviews or self-administered paper-pencil questionnaires, Computer-Assisted Self-Interviews (CASI) have been demonstrated to increase the probability of clients reporting sensitive data, e.g., sexual risk behavior [16, 17], or injecting drug use [18], and to improve completion of client intake forms resulting in significantly lower rates of missing data [19]. Benefits of CASI as perceived by patients include ensuring confidentiality and providing privacy, minimizing socially desirable responses, and helping to avoid perceived negative judgment [20]. The use of Audio CASI (ACASI) allows respondents to listen to each item and response set in their own language, which facilitates the completion and evaluation of questionnaires [21] and is of particular benefit to illiterate patients.

While ACASI has been used in various clinical settings [22–25], no research with refugees has been conducted yet. Feasibility of ACASI has been demonstrated in clinical settings including with patients with mental health symptoms [18, 26, 27] but studies have so far failed to take the usability of ACASI into account. In summary, there is no study to date that examined the feasibility of ACASI in a clinical setting with a population of traumatized refugees. Moreover, the view of health professionals with regard to usability of ACASI in traumatized refugees has never been assessed.

At the Outpatient Unit for Victims of Torture and War at the Department of Psychiatry and Psychotherapy (University Hospital Zurich, University of Zurich), we developed the Multi-Adaptive Psychological Screening Software (MAPSS). This software aims to facilitate self-report-based standardized multi-lingual mental health assessment and research. Given the gaps in the literature, the aims of this study were: (1) to assess the reliability of MAPSS as compared to paper-pencil assessments, (2) to evaluate the feasibility of the user interface of MAPSS in a clinical setting among traumatized refugees and (3) to test the usability of the administrator interface MAPSS among health professionals.

## Methods

### Participants and procedure

**Fig. 1:**
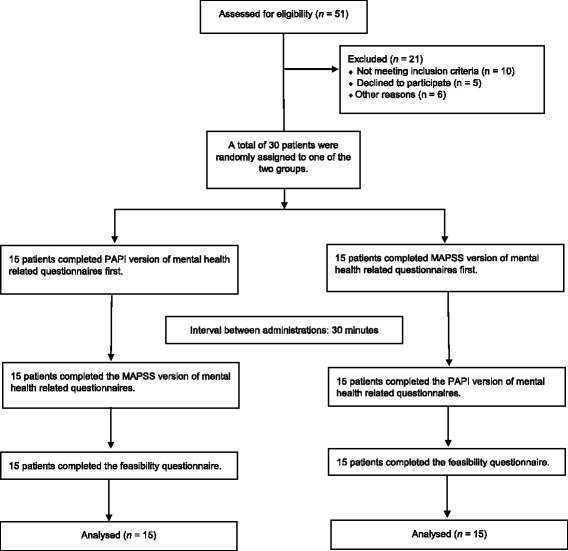
Flow diagram

A randomized cross-over design was used in the current study. A total of *N* = 30 treatment-seeking patients (see Fig.1) were enrolled from December 2015 to March 2016 in the outpatient clinic to test the feasibility of MAPSS. The study procedures were approved by the Ethics Committee of the Canton of Zurich (KEK-ZH-Nr. 2011–0495). Inclusion criteria were speaking either Tamil, Arabic, Farsi or Turkish and being able to use a touchscreen. All participants were currently undergoing treatment for their trauma-related psychosocial problems. All participants were provided study information in their native language and gave written informed consent. Of the included sample, 23% (*n* = 7) were women. The mean age was 50.07 years (*SD* = 8.65, range = 28–64). Further descriptives are shown in Table 1.

**Table 1:** Socio-demographic characteristics and previous touchscreen experience

	Condition A	Condition B	
	(*n* = 15)	(*n* = 15)	
	*M* (*SD*)	*M* (*SD*)	*t*
Age (years)	50.73 (7.80)	49.4 (9.66)	0.42
	*N* (%)	*N* (%)	*c* ^2^
Sex			1.677
Male	10 (67)	13 (87)	
Female	5 (33)	2 (13)	
Country of origin			3.040
Afghanistan	1 (7)	0	
Iraq	1 (7)	0	
Sri Lanka	1 (7)	1 (7)	
Turkey	12 (79)	13 (86)	
Sudan	0	1 (7)	
Language			1.040
Turkish	12 (79)	13 (86)	
Arabic	1 (7)	1 (7)	
Farsi	1 (7)	0	
Tamil	1 (7)	1 (7)	
Education			4.986
Attended primary school	2 (13)	3 (20)	
Completed primary school	5 (34)	2 (13)	
Attended high school	1 (7)	3 (20)	
Completed high school	3 (20)	5 (34)	
Went to technical college	2 (13)	2 (13)	
Postgraduate degree	2 (13)	0	
Previous touchscreen experience			3.628
None at all	3 (20)	4 (26)	
A little	4 (27)	7 (47)	
Quite a bit	3 (20)	3 (20)	
A lot	5 (33)	1 (7)	

There are no significant differences between conditions A and B

In the first part, participants were given a description of the procedures and were randomly assigned to two groups starting either with a paper-pencil-interview with a therapist in the presence of a qualified interpreter (PAPI; condition A) or MAPSS administration on a touchscreen device (condition B). Each participant completed health related questionnaires in the respective condition, with a 30-min break interval between administrations. Between the two conditions, the question order was randomized in each group in order to mitigate potential carry over effects. After the cross-over and second administration, all participants completed the questionnaires on feasibility on the touchscreen device. Additionally, duration of completion was measured in both modes. Participants received 20 Swiss francs (CHF; approximately $USD 20) for their participation.

In a second part, to test the usability of MAPSS among different professionals, three experienced health professionals (2 psychologists, 1 psychiatrist), one psychology student and one member of the administrative staff participated in this study. They performed three tasks within MAPSS: (1) creating a questionnaire, (2) creating a personalized user account and (3) conducting an interview. Following this, they completed the usability questionnaires.

### Measures

#### Multi-adaptive psychological screening software (MAPSS)

MAPSS was developed and programmed in cooperation with clinical scientists, based on our research group’s earlier experiences with MultiCASI [28]. It consists of both an administrator and a user interface and adapts automatically to different operating systems and devices such as tablets and smartphones. The administrator operates on an interface with three sections: (1) the editor section in which the administrator can add instructions, items, questionnaires, a variety of reply sets, and rules for skipping items and can upload audio files; (2) the interview section in which the administrator can create questionnaire sets, define their order, choose a language and create a link either for a personalized or an anonymous interview, and (3) the results section in which the administrator can organize and automatically export results for statistical analysis and show diagnostic reports. The interviewee operates on a surface on which items previously entered by the administrator are displayed in the interviewee’s language of choice. Depending on the user’s preferences, items can also be presented in spoken language by activating an audio file.

#### Questionnaires

All instruments in this study were translated and back-translated by accredited translators in accordance with gold standard translation practices [29]. Discrepancies were rectified jointly by the research team and independent translators who were experienced in working with health-related instruments.

#### Mental health related questionnaires

Posttraumatic stress disorder symptoms in the past month were assessed by a version of the Posttraumatic Diagnostic Scale based on DSM-5 (PDS, items 1–20; [30]). Symptoms of depression in the last week were measured using the 15-item subscale of the Hopkins Symptom Checklist-25 (HSCL-25, items 11–25; [31]). Overall quality of life was assessed with the first item of the EUROHIS-QoL questionnaire [32].

#### Feasibility questionnaire

Feasibility was measured by an 18-item questionnaire developed for this study based on earlier feasibility studies [33–35]. The questions covered technical feasibility, privacy and trustworthiness, perceived data safety, emotional reaction, interest and motivation, and general feasibility to indicate participants’ acceptance of MAPSS in comparison to PAPI (e.g., “Which mode is best for answering sensitive questions?” for detailed list of items see Table 3). The response format was a five-point Likert scale (1 = PAPI, 2 = Rather PAPI, 3 = Neither PAPI nor MAPSS, 4 = Rather MAPSS, 5 = MAPSS).

#### Usability questionnaires for the administration interface

The System Usability Scale (SUS; [36]) is a 10-item-scale for the subjective assessments of usability including the aspects effectiveness, efficiency and satisfaction. SUS is a standard instrument in user experience research and has proven good reliability and validity even in small samples [37–39]. Example items are *“I found the system unnecessarily complex”* and *“I thought the system was easy to use”* and are rated on a five-point Likert scale, 1 “strongly disagree” to 5 “strongly agree”. The SUS score can reach a value between 0 and 100. Each Item is coded with a value from 0 to 4 and all values are summed up (gaining a value between 0 and 40) and are multiplied by the factor 2.5 adding up to the SUS score. A score of 100 corresponds with a perfect system without usability-problems, 80 stands for good to excellent usability, 60–80 are mediocre and a SUS score under 60 indicates massive usability problems.

The AttrakDiff™ [40] is a validated online instrument examining the perceived character of interactive products (in this case the MAPSS software). It is based on the empirically well documented theoretical working model of Pragmatic and Hedonic Quality and is widely used in user experience research [41]. It consists of 28 seven-step items in the format of semantic differentials, the poles of which are opposite adjectives (e.g. “confusing - clear”, “unusual - ordinary”, “good - bad”). It measures the following dimensions: (1) Pragmatic Quality (PQ): the perceived quality of a product to gain the desired goal by offering practical functions, (2) Hedonic Quality – Stimulation (HQ-S): the ability of a product to improve one’s knowledge and skills, (3) Hedonic Quality – Identity (HQ-I): the ability of a product to communicate self-serving information to others and (4) Attraction (ATT): overall rating of the product [41]. Internal consistency was 0.73–0.90 for the subscales [40].

### Data analysis

Data analyses were performed using IBM SPSS 23. Effects of the mode of administration were examined by different strategies. The intraclass correlations (ICC) of the scale sum scores in both conditions were computed. To support measurement equivalence, ICCs of > .70 are required for group comparisons [42]. Paired-samples *t*-tests were conducted to compare the HSCL, PDS, and EUROHIS-QoL response behavior between the two administration modes PAPI and MAPSS in conditions A and B. For the feasibility questionnaire mean, standard deviation and range were computed for each item. The AttrakDiff™ was automatically analyzed by the official administration homepage on the basis of mean values and confidence intervals [43].

## Results

Table 1 shows the demographic characteristics of the study participants. The mean age was 50.73 (*SD* = 7.77, range: 35–64) years in the condition A group and 49.40 (*SD* = 9.66, range: 28–60) years in the condition B group. Approximately 21% of the patients had no experience using a touchscreen. The questionnaires in both modes were administered in Turkish, Arabic, Farsi, and Tamil. No statistically significant differences were observed for demographic characteristics and touchscreen experience between the two groups.

### Evaluation of the reliability and feasibility of MAPSS (user interface) among traumatized refugees

#### Measurement equivalence

A high degree of reliability was found between the PDS scores administered in both modes under study (condition A and B). The average measure ICC was .998 with a 95% confidence interval from .997 to .999 (*F*(29, 29) = 633.83, *p* < .001). The ICC did not differ between the two modes PAPI (ICC = .997 [.992, .999], *F*(14, 14) = 375.65, *p* < .001) and MAPSS (ICC = .998 [.995, .999], *F*(14, 14) = 660.07, *p* < .001).

A high degree of reliability was found between the HSCL scores administered in both modes under study (condition A and B). The average measure ICC was .996 with a 95% confidence interval from .991 to .998 (*F*(29, 29) = 239.65, *p* < .001). The ICC did not differ between the two modes PAPI (ICC = .993 [.979, .998], *F*(14, 14) = 140.78, *p* < .001), and MAPSS (ICC = .998 [.995, .999], *F*(14, 14) = 140.78, *p* < .001).

Paired-samples *t*-tests were conducted to compare the HSCL-25, PDS, and EUROHIS-QoL response behavior between the two administration modes PAPI and MAPSS in conditions A and B. Results indicated no significant differences in response behavior between the two administration modes PAPI and MAPSS in both condition groups for the PDS and the EUROHIS-QoL. Significant differences were found between the two administration modes PAPI and MAPSS in both condition groups for the HSCL-25. Results are shown in Table 2.

**Table 2:** Mean scores and equivalence test between PAPI and MAPSS mode for conditions A and B

	PAPI (*n* = 15)		MAPSS (*n* = 15)				
	*M*	*SD*	*M*	*SD*	*t*	*p*	*n*
Condition A							
HSCL-25	36.6	8.63	35.53	7.86	2.98	0.01	15
PDS	30.4	10.66	30.13	10.26	1.17	0.26	15
EUROHIS-QOL	3.13	1.30	3.00	1.25	1.47	0.16	15
Condition B							
HSCL-25	35.73	11.93	34.93	11.50	2.57	0.02	15
PDS	25.87	13.02	25.40	12.44	1.83	0.09	15
EUROHIS-QOL	3.40	1.60	3.40	1.4	0.00	1.00	15

*PAPI* paper-pencil-interview, *MAPSS* Multi-Adaptive Psychological Screening Software, *HSCL-25* Hopkins Symptom Checklist-25, Depression Subscale (Items 11–25), *PDS* Posttraumatic Diagnostic Scale, *EUROHIS-QOL* EUROHIS-Quality of Life (Item 8)

#### Feasibility

Twenty-five participants (83%) had no technical problems (difficulty using the touchscreen, difficulty finding the right keys to push) using the touchscreen, whereas five (17%) had trouble making corrections to their answers. Five participants (17%) used the voice output all the time, four participants (13%) some of the time and 19 participants (63%) never used it (possible answers “most of the time” and “very little of the time” were not selected by any participant). One of the participants who used the voice output all the time stated the computer voice went too slow. Twenty-four participants (80%) stated that the touchscreen mode was user-friendly and one fifth did not think so. Sixteen participants (63%) stated that the touchscreen mode made it easier for them to answer the questions, eight (27%) that the touchscreen made it rather easier and three participants (10%) that the PAPI made it easier to answer questions. Fourteen participants (47%) felt that the touchscreen mode provided more privacy, five participants (16%) felt the same for the PAPI mode, whereas 11 participants (37%) rated MAPSS and PAPI equally regarding privacy. Sixteen participants (53%) reported that the touchscreen mode encouraged more honest and truthful answers in comparison to the PAPI mode (47%). The majority (25 participants, 83%) reported thinking their personal data was safer in touchscreen mode and for five participants (17%) neither of the administration modes guaranteed higher data safety. Sixteen participants (53%) stated that it was easier to disclose personal information using the touchscreen, 11 participants (37%) found it rather easier to disclose personal information using the touchscreen and three participants (10%) thought it was easier in the PAPI mode. Twenty-five participants (83%) felt more comfortable answering sensitive questions using the touchscreen, whereas five participants (17%) felt more comfortable in the PAPI mode. All participants agreed that they enjoyed using the touchscreen to answer the questionnaires and that they would use it again during their treatment. In an overall rating 21 (70%) of the users reported that they preferred the touchscreen mode with MAPSS to answer questionnaires, one participant (3%) preferred the PAPI mode and to eight (27%) it did not matter which mode they used. Further questions concerning the comparison between the two modes on different dimensions are shown in Table 3.

**Table 3:** Feasibility of the MAPSS software on the touchscreen device in comparison to PAPI (*N* = 30)

	*M*	*SD*	*range*
Which mode made it easier to answer the questions?	4.33	1.21	1–5
Which mode made it easier to correct answers?	2.37	0.89	1–3
Which mode provides more privacy while answering questions?	3.43	1.30	1–5
In which mode, do you think your personal data is safer?	4.67	0.76	3–5
Which mode is best for answering sensitive questions?	3.33	1.12	1–5
Which mode rather encourages honest and truthful answers?	4.53	0.51	4–5
Which mode made it easier to disclose personal information?	4.23	1.19	1–5
Which mode is more stressful to complete?	2.67	1.09	1–4
In which mode, did you feel more comfortable reporting your answers?	4.17	1.49	1–5
Which mode is more interesting to use?	3.60	1.10	1–5
Which mode made it easier to stay concentrated?	3.73	1.72	1–5
Which mode is faster to complete?	4.83	0.38	4–5

Answer set based on a Likert scale: *1* PAPI, *2* Rather PAPI, *3* Neither PAPI nor MAPSS, *4* Rather MAPSS, *5* MAPSS

#### Duration of assessment

In both conditions, the time taken to complete the questionnaire was significantly shorter in the MAPSS mode than in the PAPI mode. In condition A, MAPSS (M = 00:09:00, SD = 00:02:14) was significantly more quickly completed by the participants than PAPI (M = 00:24:45, SD = 00:05:21), *t* = −16.318, *p* = .000, *n* = 15), with Cohen’s effect size (Cohen, 1992) *r* = 1.03 indicating a very large effect. In condition B MAPSS administration (M = 00:08:48, SD = 00:02:19) was also was significantly faster than PAPI (M = 00:24:10, SD = 00:05:16), *t* = −17.376, *p* = .000, *n* = 15), with Cohen’s effect size *r* = 1.03, again indicating a very large effect. This was also the case for the participants who used the voice output in MAPSS for every item, t(4) = −6.81, *p* < .005.

### Evaluation of the usability of MAPSS (administrator interface) among professionals

**Fig. 2:**
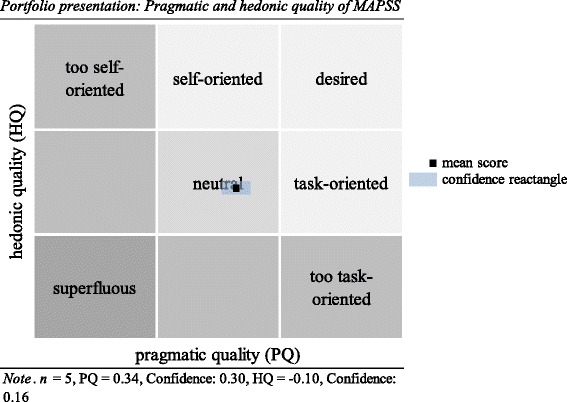
Portfolio presentation: Pragmatic and hedonic quality of MAPSS

**Fig. 3:**
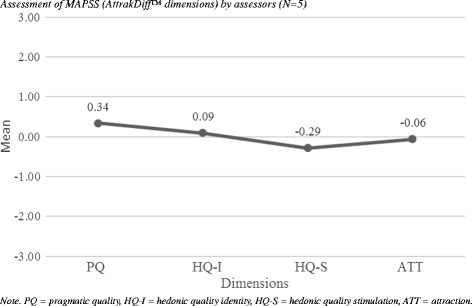
Assessment of MAPSS (AttrakDiff™ dimensions) by assessors (*N* = 5)

The Total SUS Score was 87.5 (*SD* = 10.20), CI 95% [83, 92] indicated an excellent usability rating [37]. Figure 2 (portfolio presentation) shows the mean values for PQ and HQ (averaged HQ-I and HQ-S values). For both dimensions 95% CIs were calculated and then combined into a confidence rectangle. The size of the so-called confidence rectangle shows how consistently the users rated MAPSS. A small rectangle means that the subjects rated the product very similarly, a large rectangle shows that the subjects judged the product differently. The AttrakDiff™ yielded reliable results, the different professional users (psychologist, psychiatrist, student and administrative staff) being consistent in their usability rating of MAPSS. Both the HQ and PQ were rated as medium by the professionals, indicating a good balance between self- and task-orientation. Figure 3 (diagram of average values) shows the average values of the AttrakDiff™ dimension. PQ and HQ-I of MAPSS lie slightly above average, whereas HQ-S and ATT lie slightly below average. MAPSS was rated highest on PQ in comparison to the other dimensions. Words the professionals selected to describe MAPSS were: straightforward (PQ), connective and integrating (HQ-I), captivating and challenging (HQ-S), and attractive and appealing (ATT).

## Discussion

The aim of this study was to test the reliability, feasibility and usability of MAPSS, a newly developed Audio Computer Assisted Software administered via touchscreen device, among traumatized refugees and health care professionals in a clinical setting.

The administration via MAPSS took significantly less time than the paper-pencil interview, was rated less stressful to use and more likely to encourage honest and truthful answers. A high degree of reliability was found between the symptom scores administered in both modes under study (MAPSS vs. paper-pencil). Symptoms scores in all measures were slightly lower when assessed via MAPSS as compared with PAPI, differences, however, were only significant with regard to depression. We interpret this finding in the sense of a self-presentation bias leading to more pronounced symptom presentation in front of therapist and interpreter as compared to a tablet. This interpretation is in line with earlier studies, which found lower social desirability bias in ACASI assessments versus face-to-face interviews [20]. In addition, interpreter-related misunderstandings leading to inaccurate results in case of PAPI cannot be ruled out, particularly in view of the different language backgrounds and interpreters included in the study. The results of the feasibility questionnaire (user interface) indicated a high acceptance of MAPSS and preference in comparison to PAPI among the study sample. The MAPSS software was shown to be highly accepted among the participants and all participants agreed to use MAPSS again during their treatment. 30% of the participants used the voice output in addition to the written items presentation. These results indicate that the voice output may be an essential feature and a strong asset of MAPSS, particularly for participants with limited literacy.

The usability of MAPSS (administrator interface) was rated as excellent by different professionals (psychologists, psychiatrist, student, administrative staff), indicating that MAPSS is quick to learn and easy to use. Professional administrators gave the usability of the administration interface of MAPSS a high rating particularly on the pragmatic quality dimension, meaning that the software is practical in achieving the desired goal to administer and analyze questionnaires in different languages. Professionals emphasized the informative content of the software and described it as straightforward, connective and integrating, attractive and appealing. However, both the hedonic quality and attraction were rated slightly below average. This could be rooted in the targeted functional purpose of the software, which does not aim at pleasing administrators with its functions or to broaden the personal skills of the user.

Future studies should use and evaluate CASI/ACASI in larger samples, testing it for population-level epidemiological surveys, systematic screenings and needs assessments, particularly in low resource settings. Studies should explicitly address the aspect of cost-effectiveness and explore the potential clinical and economic benefits of implementing MAPSS as a subsidiary tool within standard diagnostic procedures. Additional examinations, e.g. by qualitative methods, should provide more insight on its use and its potential value in collecting sensitive trauma exposure data where shame, stigma or the anticipation of social sanctioning may be prohibitive in a conventional interpreter-based setting.

This study has several limitations. First, the small sample size limits the generalizability of the results. Second, we did not analyze the influence of educational level on the ability to use a touchscreen device. Yet, it has been shown, that education has no influence on the ability to use a touchscreen [44]. For this study, we felt that previous touchscreen experience was a sufficient measure to account for possible differences in the participants’ ability to use a tablet. Third, the interval between the two administration modes was only 30 min, which might have caused carry-over effects [45]. To account for that, we changed the order of the questions between the two administration modes. A longer interval of, e.g., several days, may include a change in the patients’ condition or discourage them to participate in the second administration.

## Conclusion

Our results suggest that MAPSS administered via touchscreen device demonstrates good reliability, is fast to implement and well accepted by both patients and professionals, underlining its potential as an advantageous alternative to paper-pencil or interpreter-based interviews in multi-lingual settings. In addition, the automatic data export saves resources, reduces missing data and possible errors in data input. Accordingly, MAPSS could also be useful for mental health screenings in refugee camps and other low-resource settings, and MAPSS could be a very helpful tool to perform health screenings very early in an asylum process or in epidemiological studies. In addition, not only specialized psychiatric institutions could benefit from MAPSS but also general practitioners or other professionals who do not have the possibility to use interpreters. Although the use of qualified interpreters remains indispensable in the therapeutic process, MAPSS may represent a meaningful tool in order to allocate limited resources to those most in need.

## Abbreviations

ACASI: Audio computer-assisted self-interview software
ATT: Attraction
CASI: Computer-assisted self-interviews
CI: Confidence interval
HQ-I: Hedonic quality
HQ-S: Hedonic quality – stimulation
HSCL: Hopkins symptom checklist
ICC: Intraclass correlations
MAPSS: Multi-adaptive psychological screening software
PAPI: Paper-pencil-interview
PDS: Posttraumatic diagnostic scale
PQ: Pragmatic quality
PTSD: Posttraumatic stress disorder
SD: Standard deviation
SUS: System usability scale
UNHCR: United Nation high commissioner for refugees
